# Methodologies on estimating the energy requirements for maintenance and determining the net energy contents of feed ingredients in swine: a review of recent work

**DOI:** 10.1186/s40104-018-0254-0

**Published:** 2018-05-16

**Authors:** Zhongchao Li, Hu Liu, Yakui Li, Zhiqian Lv, Ling Liu, Changhua Lai, Junjun Wang, Fenglai Wang, Defa Li, Shuai Zhang

**Affiliations:** State Key Laboratory of Animal Nutrition, Ministry of Agriculture Feed Industry Centre, China Agricultural University, Beijing, 100193 China

**Keywords:** Heat production, Ingredients, Maintenance, Net energy, Prediction equations, Validation

## Abstract

In the past two decades, a considerable amount of research has focused on the determination of the digestible (DE) and metabolizable energy (ME) contents of feed ingredients fed to swine. Compared with the DE and ME systems, the net energy (NE) system is assumed to be the most accurate estimate of the energy actually available to the animal. However, published data pertaining to the measured NE content of ingredients fed to growing pigs are limited. Therefore, the Feed Data Group at the Ministry of Agricultural Feed Industry Centre (MAFIC) located at China Agricultural University has evaluated the NE content of many ingredients using indirect calorimetry. The present review summarizes the NE research works conducted at MAFIC and compares these results with those from other research groups on methodological aspect. These research projects mainly focus on estimating the energy requirements for maintenance and its impact on the determination, prediction, and validation of the NE content of several ingredients fed to swine. The estimation of maintenance energy is affected by methodology, growth stage, and previous feeding level. The fasting heat production method and the curvilinear regression method were used in MAFIC to estimate the NE requirement for maintenance. The NE contents of different feedstuffs were determined using indirect calorimetry through standard experimental procedure in MAFIC. Previously generated NE equations can also be used to predict NE in situations where calorimeters are not available. Although popular, the caloric efficiency is not a generally accepted method to validate the energy content of individual feedstuffs. In the future, more accurate and dynamic NE prediction equations aiming at specific ingredients should be established, and more practical validation approaches need to be developed.

## Background

Most pigs are fed diets based on cereal (corn, wheat, barley, etc.) and protein sources (soybean meal, etc.). However, less-expensive, alternative feed ingredients either rich in dietary fiber or protein, such as corn by-products and oil-seed meals, have been increasingly included in swine diets in order to decrease feed costs [1]. In addition, using the net energy (NE) system may help to reduce feed cost because it takes the heat increment (HI) into consideration. Therefore, it is a more precise evaluation of the true energy value of the feed compared to the metabolizable energy (ME). In most situations, the NE content of ingredients is predicted from equations. The most widely used prediction equations were developed more than 20 years ago and include NE prediction equations for growing pigs [2], fattening pigs [3], maintenance-fed adult sows [4], and growing boars [5]. These studies covered wide range of ingredients and demonstrated that NE contents of ingredients or diets can be estimated based on their chemical compositions or digestible nutrients. Nevertheless, some highly processed ingredients (or by-products) that have become more available nowadays (corn germ meal, cottonseed meal, rice bran, etc) were not included. Therefore, these prediction equations may not be suitable for these new feedstuffs.

Nine open-circuit respiration chambers were established at Ministry of Agriculture Feed Industry Centre (MAFIC) according to the design of van Milgen et al. [6]. Since 2012, many research projects have been conducted in MAFIC to determine the maintenance energy requirements for pigs and the NE content of individual ingredients. All data were determined using the same methods and procedures [1, 7–14]. The objective of this review is to summarize the NE-related work conducted at MAFIC and compare these results with those from other research groups on methodological aspect, which included estimating the energy requirements for maintenance as well as the determination, prediction, and validation of the NE content of various ingredients.

### Some concepts about energy

**Fig. 1:**
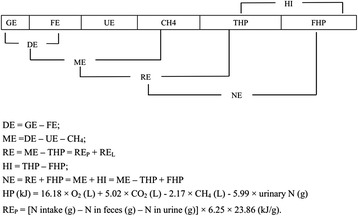
Scheme of energy utilization in pigs. The utilization of energy in a feed by pigs is a multi-level system. GE, gross energy; DE, digestible energy; ME, metabolizable energy; RE, retained energy; NE, net energy; FE, fecal energy; UE, urinary energy, THP, total heat production; FHP, fasting heat production; HI, heat increment

When measuring NE by the indirect calorimetry method, heat production (HP) can be calculated from gas exchanges (open-circuit respiration chambers) and the urinary nitrogen according to Brouwer [15]. The utilization of the energy in a feed by pigs is a multi-level system (Fig. 1). The ME can be partitioned into two parts: NE and HI. HI is the heat produced during the process of nutrient ingestion, digestion, and metabolism, and is thought to be useless [16]. However, it is difficult to separate the HI from total heat production (THP), and HI is typically calculated as THP minus NE requirements for maintenance (NE_m_). The NE_m_ also cannot be determined directly, and fasting heat production (FHP), which is measured in the fasted animal, is frequently used to estimate the NE_m_ [2, 5, 17]. As a result, HI is calculated as THP minus FHP. In growing animals, NE is usually estimated as the sum of NE_m_ and retained energy (RE), and RE is usually evaluated as ME intake minus THP. In growing pigs, RE corresponds to the sum of RE as protein (RE_P_) and RE as lipid (RE_L_). The RE_P_ is typically calculated using nitrogen retention (g) = N × 6.25 × 23.86 (kJ/g) [18] and RE_L_ is calculated as the difference between RE and RE_P_ (Fig. 1).

### Information on intake and output for pigs

Modern pig production contributes to many environmental problems that relate to excretion from pigs, especially in areas with highly intensive production systems. Therefore, measurement of intake and output data is very useful for the global design of manure management systems for swine [19].

In our NE trials, the inputs including feed intake, gross energy (GE) intake and O_2_ consumption as well as the outputs including feces, urine, CO_2_, CH_4_, and HP have been summarized according to diet characteristics (Table 1). Although pigs were fed in crates and feed intake was lower than ad libitum, these data may still be useful as a reference for the actual production situation. Almost half of the GE consumed is used for HP and only one third of the GE is retained. Approximately 14% of the GE is excreted in the feces, 3% of the GE is excreted in the urine and 0.7% of the GE is excreted as methane.

**Table 1:** Information on intake and output for pigs^a^

Diets	*n*	Mean BW, kg	Intake		Output								Gas exchange, L/d			Digestibility, %	
			Feed, kg/d	GE, MJ/d	Feces		Urine		CH_4_ energy, MJ/d	HP, MJ/d			O_2_	CH_4_	CO_2_	DM	GE
					Weight, g/d	GE, MJ/d	Volume, L/d	GE, MJ/d		THP	FHP	HI					
Corn-soybean meal	30	45.53	1.46	24.31	380.4	2.57	2.64	0.57	0.19	12.73	7.66	5.07	606.4	4.9	660.8	73.9	89.4
Oil-seed meals^b^	66	44.78	1.45	24.51	576.6	3.80	3.19	0.81	0.14	12.26	7.50	4.76	587.8	3.6	619.6	60.2	84.5
Fibrous ingredients^c^	24	44.70	1.54	24.62	766.6	4.47	2.33	0.74	0.16	12.04	7.22	4.82	593.8	4.1	634.1	50.2	81.8
Corn DDGS	36	47.08	1.68	28.09	622.1	4.09	3.08	1.02	0.26	13.69	7.78	5.91	651.7	6.6	735.5	63.0	85.4
Mean^d^	168	45.07	1.50	25.22	558.2	3.62	2.89	0.78	0.18	12.61	7.53	5.08	604.8	4.5	654.9	62.8	85.6

^a^Data from NE trials conducted at MAFIC; pigs were fed in crates with a feed intake lower than ad libitum; GE, gross energy; HP, heat production; THP, total heat production; FHP, fasting heat production; HI, heat increment

^b^Includes rapeseed meal, peanut meal, sunflower meal, and cottonseed meal

^c^Includes rice bran, corn germ meal, corn gluten feed, and wheat bran

^d^Excluding corn-SBM, oil-seed meals, high-fiber meals, and corn DDGS but including wheat and corn diets

### Estimation of the energy requirements for maintenance

Estimation of the energy requirement for maintenance is very important when conducting research on energy metabolism, which will influence the absolute NE value of a feedstuff [8, 20]. The energy requirements for maintenance can be expressed as ME (ME_m_) or NE (NE_m_). The choice of methodologies and measuring conditions are very important for estimating these energy values.

**Fig. 2:**
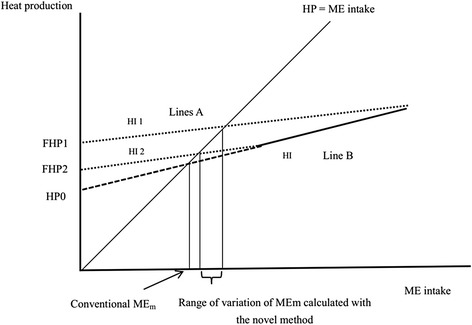
Relationship between heat production and metabolizable energy (ME) intake. HP_0_ = Extrapolated total heat production at zero energy intake. FHP 1 corresponds to the FHP measured with animals receiving the highest feed level and FHP2 corresponds to the FHP measured with animals receiving the lowest feed level. The slope of line B = HI:ME. The complement of the slope of line B is k_g_ (NE:ME). The slope of line A = (HIi:ME). The complement of the slope of line A is k_m_ (NE_m_: ME), k_m_ > k_g_ (adapted from Labussière et al. [23]; Noblet and van Milgen, [20]). FHP: Fasting heat production; HI: Heat increment; k_m_: Efficiencies of energy utilization for maintenance; k_g_: Efficiencies of energy utilization for growth; ME_m_: Metabolizable energy requirements for maintenance; NE: Net energy; NE_m_: Net energy requirements for maintenance

**Fig. 3:**
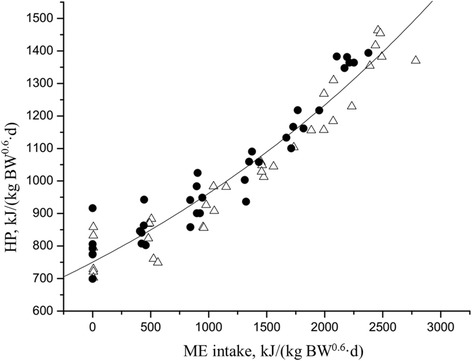
Exponential relationship between heat production and metabolizable energy (ME) intake for all pigs. HP = 749 × e ^(0.0002 × ME intake)^, *R*^*2*^ = 0.91, *P* < 0.001. Data are from growing (●), and finishing (△) pigs (Zhang et al. [8]). HP: heat production

The NE_m_ cannot be determined directly but there are mainly 2 methods which have been used to estimate NE_m_. Firstly, the FHP can be measured directly in fasted animals and it is then used as an estimate of NE_m_ [2, 5, 17]. This method is affected by the length of fasting, previous feeding level, and differences in activity between fasting and fed status [7, 8, 21]. Additionally, FHP is affected by both the ATP requirement at the cellular level and heat produced from the generation of ATP from body nutrient stores [22]. The second approach for estimating NE_m_ which has been widely used in the past is the regression method, in which THP is calculated through extrapolating HP measured at different ME intake levels to zero ME intake (HP_0_). The commonly used regression models include linear model and curvilinear model. For linear regression [5, 16], the hypothesis is that the HP increased linearly with the ME intake. However, this method has great limitations. Firstly, the ME intake levels selected for HP extrapolating typically range from 60% to 100% of ad libitum intake, thus there is a lack of information on the relationship between HP and ME intake below the energy requirements for maintenance [8, 20]. In this method, the efficiencies of energy utilization for maintenance (k_m_) and growth (k_g_) are assumed to be constant. However, recent research reported that this traditional assumption may be incorrect [8, 9, 20, 23]. Secondly, the FHP value measured throught direct method is variable and is affected by the previous feeding level [8, 9, 23, 24]. These authors also reported that the HP_0_ was lower than the actual measured FHP value (Fig. 2). The curvilinear regression model to estimate the NE_m_ was proposed at MAFIC [8], which uses exponential regression between HP and a wide range of ME intakes both above and below energy requirements for maintenance (Fig. 3). In this method, the Y-intercept was thought to be the NE_m_ and takes into account the difference between k_g_ and k_m_ as well as the effect of previous feeding level on FHP. However, both the linear and curvilinear models provide only one estimate of NE_m_, while pigs fed at different feeding levels have different NE_m_.

The ME_m_ was estimated by assuming that the HP is equal to ME intake at maintenance. Therefore, based on the NE_m_ estimation, the ME_m_ was conventionally calculated by solving the regression quations (linear regression model, HP = a + b ME intake; exponential regression model, HP = a e^b×ME intake^). A novel method to estimate ME_m_ was proposed by Labussière et al. [23], in which the estimated ME_m_ ranged from the ME_m_ calculated at the lowest feeding level to ME_m_ calculated at the highest feeding level (Fig. 2). ME_m_ is affected by feeding levels and estimated ME_m_ decreases in growing pigs when ME intake is reduced [23]. Several hypotheses have been suggested to explain the results, including the effect of the gastrointestinal tract size [6, 25], or the changed muscle protein turnover rate [26].

**Fig. 4:**
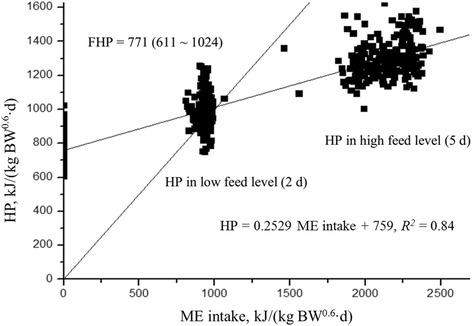
Regression of heat production on metabolizable energy (ME) intake. 16 experiments, 34 diets, 26 ingredients, 204 replications with 408 heat production and 204 fasting heat production data were included in the figure. FHP: Fasting heat production; HP: heat production

In our NE trials, where FHP was considered as the HP measured for 8 h after 31 h fasting from the last meal, the HP_0_ (759 kJ ME/ (kg BW^0.6^·d)) linearly regressed on HP and ME intake was in the range of the FHP (611 to 1,024 kJ ME/ (kg BW^0.6^·d), a few of extreme outliers existed, and most FHP values ranged around 700 to 800, Table 2 and Fig. 4). The following regression equation was established based on all data in our NE trials: HP = 0.2529 ME intake + 759 (*R*^2^ = 0.84). Consequently, the ME_m_ = 1,016 kJ ME/ (kg BW^0.6^·d).

**Table 2:** Comparison of extrapolated total heat production at zero energy intake (HP_0_) and FHP.^a^ All works listed were conducted in MAFIC

No.	Authors	Experiments (diets)	HP_0_	FHP	Range of FHP
1	Liu et al. (2015) [10]	Complete diets	768	781	605–901
2	Li et al. (2015) [1]	Corn DDGS diets	783	795	611–971
3	Li et al. (2017) [13]	Rapeseed meal	768	789	692–1,024
4	Li et al. (2018) [14]	Five ingredients	717	770	615–973
5	Li et al. (unpublished)	Corn	737	716	648–869
6	Li et al. (2017) [12]	Rapeseed meal	798	775	651–866
		Summary	759	771	611–1,024

^a^Includes 6 experiments with 34 diets and 26 ingredients, 204 replications, 408 heat production and 204 fasting heat production

The estimated energy requirements for maintenance among different research projects are shown in Table 3. These values depend on the methods used, growth stage, conditions of measurement (e.g. procedure, activity), feeding levels and the composition of the diet; they should then be interpreted with caution and are not directly comparable. Accounting for the effect of feeding level on FHP, the measurement of FHP immediately after a fed period is highly preferable [20]. The ME_m_ (1,016 kJ ME/ (kg BW^0.6^·d), summarized in MAFIC) was very close to the value (1,020 kJ ME/ (kg BW^0.6^·d)) obtained from different breeds and variable BW [27].

**Table 3:** Comparison of the estimation of energy requirements for maintenance among different experiments^a^

Reference	Methods	BW, kg	ME_m_	NE_m_ (HP_0_)
Noblet et al. (1999) [27]	Linear regression	Variable	1,020^b^	750^c^
Labussière et al. (2011) [23]	Ranged values based on feeding levels	60–90	822–1028	608–771
Zhang et al. (2014) [8] (MAFIC work)	Linear regression	30–60	893	590
	Linear regression	60–90	815	532
	Exponential regression	30–60	973	758
	Exponential regression	60–90	921	732
Li et al. (2017) [13] (MAFIC work)	Linear regression	30–60	1,016	759
	Measured FHP	30–60	–	771

^a^Energy unit: kJ / (kg BW^0.6^·d)

^b^The average value for different breeds, variable BW

^c^Data from Noblet et al. [5]: all pigs were fed at a high FL and immediately fed a reduced FL

In conclusion, estimating the energy requirements for maintenance is a complex project. Future research should focus more on biochemical mechanisms and use new technologies to find more meaningful criteria which are representative of the maintenance condition for pigs.

### Net energy content of ingredients

Previous studies have shown that the DE and ME systems systematically underestimate the energy concentration of feeds high in starch or fat and overestimate the energy concentration of feeds high in protein or crude fibre [5, 28]. Therefore, the NE system should be the most appropriate system by now to estimate the true energy value of feed ingredient, which is theoretically independent of the feed characteristics [28–31]. Large amounts of data pertaining to the determination of NE value of ingredients were published during the past decade (Table 4). Indirect calorimetry and comparative slaughter are the two main methods used to determine the NE content of ingredients. The indirect calorimetry consists of measuring oxygen consumption, and carbon dioxide and methane production [20]. These measurements combined with the urinary energy production are then used to calculate HP based on equations from Brouwer [15]. The comparative slaughter technique, on the other hand, directly measures energy gain based on chemical analysis of samples collected after slaughter, and HP is calculated as the difference between ME intake and energy gain [20]. Due to differences in experimental procedures, the chemical composition of ingredients, animals, and errors in analysis, it is difficult to compare these two methods for a given ingredient, and determine which method should be used to estimate the true energy available to an animal. Ayoade et al. [32] reported that the NE in wheat and corn DDGS diets obtained with indirect calorimetry and comparative slaughter method did not differ. However, many other results indicated that the NE values measured by indirect calorimetry were greater than values calculated with the comparative slaughter method [33–36], which may be attributed to the fact that in studies using comparative slaughter technique, pigs were housed in a more practical facility and then can move freely with more heat production related to physical activity. Moreover, pigs in studies using comparative slaughter technique may sometimes be raised below their critical temperature with again an increased heat production. Therefore, heat production measured using comparative slaughter method may be highly increased in connection with increased maintenance energy and FHP values, while the FHP value used in indirect calorimetry procedure is obtained under minimal activity and at thermo-neutrality, which leads to the greater NE value [37, 38].

**Table 4:** Summary of research on the net energy content of ingredients conducted in the past decade (MJ/kg DM)

Authors	Ingredients	Pigs	NE	NE/ME	Methods
Hinson et al. (2009) [53]	Soybean meal	Growing	7.66	–	Comparative slaughter
		Finishing	10.08	–	
	Low-oligosaccharide soybean meal	Growing	9.14	–	
		Finishing	11.73	–	
	Glycerol	Growing	13.44	–	
		Finishing	16.98	–	
Kil et al. (2011) [34]	Soybean oil	Growing	20.19	–	Comparative slaughter
		Finishing	19.33	–	
	Choice white grease	Growing	25.05	–	
		Finishing	24.99	–	
Kil et al. (2013) [35]	Corn	Growing	9.06	–	Comparative slaughter
		Finishing	11.08	–	
Stewart et al. (2013) [54]	Soybean hulls	Growing	1.67	–	Comparative slaughter
		Finishing	4.01	–	
	Wheat middlings	Growing	4.46	–	
		Finishing	4.72	–	
Heo et al. (2014) [55]	Canola meal (*Brassica napus*)	Growing	8.80	69.8	Indirect calorimetry
	Canola meal (*Brassica juncea*)		9.80	72.6	
Graham et al. (2014) [39]	Corn DDGS-high oil (12.1% oil)^a^	Finishing	12.99	81.7	Estimating NE efficiency from a growth study
	Corn DDGS-high oil (9.6% oil)		11.85	74.7	
	Corn DDGS-high oil (9.4% oil)		11.60	71.0	
	Corn DDGS-medium oil (7.6% oil)		10.61	75.3	
	Corn DDGS-low oil (5.4% oil)		9.61	66.0	
Gutierrez et al. (2014) [36]	Corn DDGS-BPX^b^	Growing	8.78	–	Comparative slaughter
		Finishing	8.46	–	
	Corn DDGS (13.0% oil)	Growing	9.10	–	
		Finishing	11.57	–	
	High protein DDG	Growing	9.68	–	
		Finishing	9.17	–	
Liu et al. (2014 and 2015) [9, 10] (MAFIC work)	Corn	Growing	13.21	81.0	Indirect calorimetry
	Soybean meal		10.62	64.3	
	Wheat bran		7.78	71.6	
	Wheat		11.44	74.6	
	Corn DDGS-high oil (10.6% oil)		10.21	66.5	
	Canola meal		8.38	72.0	
	Cottonseed meal		7.32	72.9	
Li et al. (2017) [11] (MAFIC work)	Corn DDGS-high oil (11.2% oil)	Growing	10.47	70.0	Indirect calorimetry
	Corn DDGS-high oil (10.7% oil)		10.98	77.0	
	Corn DDGS-medium oil (7.6% oil)		10.96	70.8	
	Corn DDGS-low oil (4.7% oil)		9.49	67.7	
	Corn DDGS-low oil (3.6% oil)		9.28	69.0	
Velayudhan et al. (2015) [42]	Dry extruded expelled SBM	Growing	10.64	75.7	Indirect calorimetry
Li et al. (2018) [14](MAFIC work)	Rice bran	Growing	12.33	77.9	Indirect calorimetry
	Corn germ meal		8.75	72.4	
	Corn gluten feed		7.51	78.5	
	Peanut meal		10.75	75.3	
	Sunflower meal		6.49	67.2	
Li et al. (2017) [13](MAFIC work)	Rapeseed meal-expeller press	Growing	10.14	72.2	Indirect calorimetry
	Rapeseed meal-expeller press		11.46	80.1	
	Rapeseed meal-solvent extracted		7.98	65.3	
	Rapeseed meal-solvent extracted		9.47	75.1	
	Rapeseed meal-solvent extracted		7.91	72.7	
Li et al. (2017) [12] (MAFIC work)	Corn	Growing	12.46	78.3	Indirect calorimetry
	Soybean meal		11.34	70.2	
	Rapeseed meal-expeller press		11.71	74.7	
	Rapeseed meal-solvent extracted		8.83	76.5	

^a^Oil content as-fed basis;

^b^Corn DDGS-BPX, uncooked corn distillers dried grains with solubles

Although questionable, Graham et al. [39] developed a new method to calculate the NE value of ingredients which estimates NE based on regression analysis determined by estimating NE efficiency (NEE) from growth studies. In this method, the NE of a corn-soybean meal control diet was calculated based on literature data and then the NEE (NE intake: ADG) was calculated according to actual pig growth. The NE of the test diet was calculated based on the hypothesis that the NEE of pigs fed a test diet was equal to that of pigs fed the control diet. The NE of the test ingredient was calculated according to the percentage of the test ingredient in the test diet.

From a practical point of view, and to avoid bias in the calculation of NE for different feedstuffs, it is necessary to use similar animals and maintain these animals under similar and standardized conditions [20]. In our laboratory, the NE trials (data shown in Tables 2, 5, and 6) were conducted in the same location (Fengning, Hebei, China) and using standardized conditions, including similar animals from same breed (Duroc × Large White × Landrace, BW ranged from 30 to 60 kg), similar chambers, a constant temperature and humidity and using the same experimental procedures. In these experiments, pigs were individually housed in metabolic crates for 16 d, which included 7 d to adapt to the feed and environmental conditions. On d 8, pigs were transferred to the open-circuit respiration chambers for measurement of O_2_ consumption as well as CO_2_ and CH_4_ production. During this time, pigs were fed their diet at 2,400 kJ ME/ (kg BW^0.6^·d). Feces and urine were collected from d 9 to d 13, and HP was measured. From d 14 to d 15, pigs were fed at the maintenance requirement (ME_m_ = 893 kJ ME/ (kg BW^0.6^·d)) obtained from the results of Zhang et al. [8] in order to adapt the pigs from the fed to the fasting state. On the last day of each period, pigs were fasted and FHP was estimated as the averaged HP of 8 h after 31 h fasting. Even not quantifying physical activity, the FHP in our trials was measured under minimum expected activity (during the night, in the dark, and adjustment of the cage, etc.) and after a long period of fasting. The FHP values measured in different studies using the same procedure conducted in MAFIC close to each other (Table 2) [1, 8–12], and also close to those obtained by the INRA group according to a specific methodology or those used in the large scale NE measurements [5].

**Table 5:** Levels of ingredients in the diets^a^

Ingredients	No. of diets	Inclusion level,%	
		Max	Mean
Corn	33	97.03	66.12
Soybean meal	26	25.00	17.58
Wheat bran	5	33.00	15.40
Wheat	4	58.37	20.59
Corn DDGS	9	29.25	17.53
Rapeseed meal	11	20.00	14.68
Cottonseed meal	4	10.00	5.75
Full-fat rice bran	1	29.25	29.25
Corn germ meal	1	29.25	29.25
Corn gluten feed	1	24.38	24.38
Peanut meal	1	19.50	19.50
Sunflower meal	1	29.25	29.25
Dicalcium phosphate	33	1.20	0.89
Limestone	34	1.46	0.83
Salt	34	0.40	0.36
Vitamin and mineral premix	34	0.50	0.50
Lysine HCl	10	0.71	0.40
*DL*-methionine	8	0.14	0.09
*L*-threonine	10	0.17	0.08
*L*-tryptophan	5	0.04	0.03

^a^Includes 6 experiments, 34 diets and 26 ingredients

**Table 6:** Chemical composition of diets^a^ (as-fed basis)

Chemical composition	Minimum	Maximum	Mean
Dry matter	85.96	90.11	87.94
Crude protein	6.87	23.79	16.59
Neutral detergent fiber	8.52	24.33	14.33
Acid detergent fiber	1.56	9.59	4.52
Ether extract	2.18	5.52	3.05
Starch	31.57	63.10	43.07
Ash	2.97	5.91	4.43
Gross energy	15.48	16.90	16.12

^a^Includes 6 experiments with 34 diets and 26 ingredients

Based on the above methodologies, a series of studies were conducted in MAFIC to evaluate the NE values of 26 ingredient samples, including 2 corn samples, 2 soybean meal samples, 1 wheat sample, 1 wheat bran sample, 8 rapeseed meal samples, 1 cottonseed meal sample, 6 corn DDGS samples, 1 rice bran sample, 1 corn germ meal sample, 1 corn gluten feed sample, 1 peanut meal sample and 1 sunflower meal sample. The estimated NE values were summarized in Table 4, and our NE values were comparable with those in feeding tables by Sauvant et al. [40] or NRC [41].

### Net energy prediction equations

The NE prediction equations have been proposed for growing pigs (Just, 1982) and growing boars [5] with the latter have being widely used during the past two decades [32, 42–44].

**Fig. 5:**
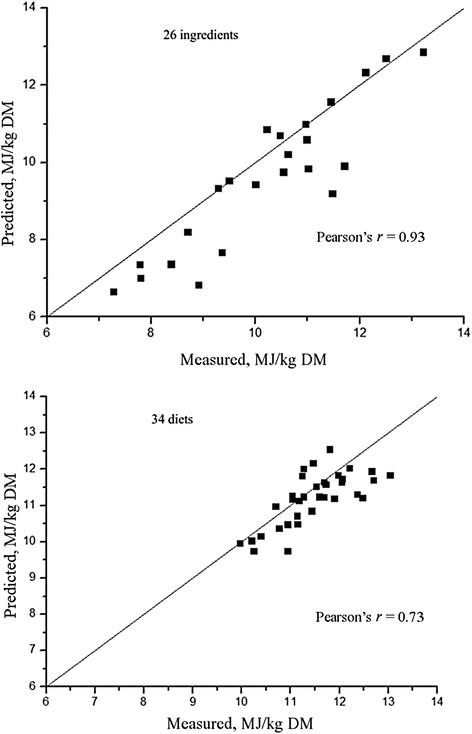
Comparison of net energy (NE) values measured at MAFIC with those predicted by equations from INRA. NE values of 26 ingredients or 34 diets measured at MAFIC or predicted based on an INRA prediction eq. (NE = (0.7 × DE) + [(1.61 × EE) + (0.48 × Starch) –(0.91 × CP)–(0.87 × ADF)]/1000 × 4.184) were illustrated. ADF: Acid detergent fiber; CP: crude protein; DE: Digestible energy; EE: Ether extract

**Fig. 6:**
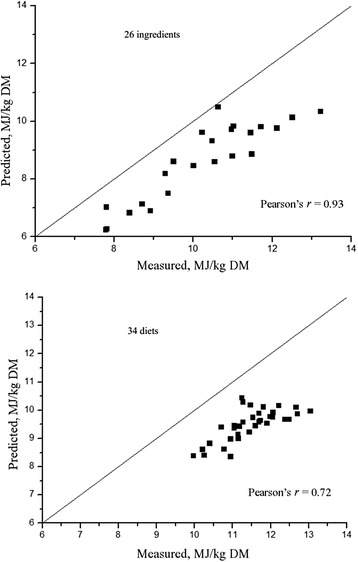
Comparison of net energy (NE) values measured at MAFIC with thosed predicted by equations by Just. NE values of 26 ingredients or 34 diets measured at MAFIC or predicted based on a prediction equation by Just [2] (NE = 0.75× ME – 1.88) were illustrated. ME: Metabolizable energy

The NE in wheat-corn DDGS diets [28] or corn-DDGS [1] measured by indirect calorimetry is similar to values obtained with prediction equations of Noblet et al. [5]. However, Kil [45] reported that NE values for diets and ingredients predicted from the equations of Noblet et al. [5] fed to growing pigs were greater than the values measured by comparative slaughter. It should be mentioned that the measured NE values were obtained with a FHP value (536 kJ/(kg BW^0.6^·d)) markedly lower than the value (750 kJ/(kg BW^0.6^·d)) used in the prediction equations of Noblet et al. [5]. There are differences in methodology used to estimate the energy requirements for maintenance. Therefore, caution should be essential when comparing the measured values with predicted values. It is also difficult to compare NE values obtained by different methods using statistical procedures. A possible reason is the relatively large standard error of the mean for the NE of ingredients compared with diets when the NE values of ingredients are calculated according to the difference procedure. The inherent problems associated with using the difference procedure were also found by Kil et al. [34]. In the present review, the NE value of 26 ingredients and 34 diets measured at MAFIC were compared with values predicted by the equations of Noblet et al. [5] and Just [2]. From the present review, the equations of Noblet et al. [5] can be used to predict the NE value of most ingredients (Fig. 5), except for those ingredients containing high fiber content, such as sunflower meal or cottonseed meal. The NE of 34 diets can be accurately predicted by the equations of Noblet et al. [5] (Fig. 5). The equation proposed by Just [2] underestimate the NE value of the 26 ingredients and 34 diets (Fig. 6). Generally, the prediction equations proposed by Noblet et al. [5] can be used to predict NE in situations where calorimeters are not available. However, more accurate NE prediction equations should be established with those equations aimed at specific ingredients such as oil-seed meals and fibrous ingredients. Future research at MAFIC will focus on such projects.

## Validation

As mentioned above, the NE value of feedstuffs can be directly determined by either comparative slaughter or indirect calorimetry technique, or can be predicted from equations. However, only a few studies have been conducted to validate these estimated NE values [46]. The recent published literatures on NE value validation have been summarized in Table 7. Among these studies, caloric efficiency is the most popular parameter used to determine whether the NE value of the ingredient was accurately estimated [12, 28, 39, 47–49]. The caloric efficiency was calculated as the daily energy intake (ADFI×dietary energy, MJ/kg) divided by ADG [39, 48, 49]. The validation approach using caloric efficiency is established based on the fact that pigs tend to consume their feed according to their energy requirements, and thus if the other nutrients such as amino acids are maintained constant, similar growth performance of pigs are expected [49, 50]. Therefore, the test ingredient was included in experimental diets in gradient levels in these validation trials, and similar caloric efficiency among the dietary treatments were expected if the assigned energy value is accurate. The NE values of tallow, soybean oil, wheat middlings, medium-oil corn DDGS, and rapeseed meal have been validated through this method [12, 28, 39, 47, 48]. Moreover, some studies used gain to feed ratio instead of caloric efficiency as a parameter to validate the estimated energy value. The underlying theories and experimental procedures of such experiments are the same as those using caloric efficiency. The NE value of flaxseed meal and canola meal have been validated through this method [46, 51].

**Table 7:** Summary of validation research published in the past decade

Authors	Ingredients	BW, kg	Methods	Experimental design^a^	Conclusion
Wu et al. (2007) [28]	Tallow	23–98	Caloric efficiency	Keep the NE to SID Lys ratio constant	The NE efficiency was not influenced by fat level, but DE and ME efficiency decreased
Eastwood et al. (2009) [51]	Flaxseed meal	32–115	Gain to feed ratio	Keep NE and SID Lys constant	The NE of flaxseed meal was correctly estimated
Montoya et al. (2010) [46]	Canola meal and full-fat canola seeds	30–60	Gain to feed ratio	Keep NE and SID Lys constant	The NE of canola meal was correctly estimated, but slightly underestimated for full fat canola seed.
Adeola et al. (2013) [47]	Soybean oil and tallow	6–25	Caloric efficiency	Keep the ME to SID Lys ratio constant	The NE of soybean oil from the 2012 NRC was accurate. NE of tallow were underestimated
De Jong et al. (2014) [48]	Wheat middlings	Nursery pigs	Caloric efficiency	Keep the SID Lys constant and isocaloric	The INRA NE of wheat midds appears to be a more accurate energy value than the ME obtained from the NRC
Graham et al. (2014) [39]	Medium-oil corn DDGS	69–126	Caloric efficiency	Keep the SID Lys constant and isocaloric	The NE of corn DDGS was accurate
Nitikanchana et al. (2015) [49]	Medium-oil corn DDGS and fat	57–124	Caloric efficiency	Keep the SID Lys constant and isocaloric	The NE of corn DDGS was overestimated and fat was underestimated
Wu et al. (2016) [52]	4 corn DDGS sources	22–115	NRC growth model; prediction error and bias	Keep NE to SID Lys ratio similar	The NE predicted by a commercial service resulted in suboptimal prediction of NE among corn DDGS sources
Li et al. (2017) [12] (MAFIC work)	Corn, SBM, rapeseed meal	36	Caloric efficiency	Keep NE to SID Lys ratio similar	The NE measured was correctly estimated

^a^DE: Digestiable energy; ME: Metabolizable energy; NE: Net energy; SID Lys: Standardized ileal digestible lysine

In the validation trials mentioned above, a key step in experimental design is to set up the constant values, usually the standardized ileal digestible lysine (SID Lys), among the dietary treatments when formulating treatment diets. In this situation, the net energy contents of the treatment diets tend to be slightly different due to the different inclusion levels of the test ingredients [39, 48, 49]. Some researchers then adjusted the energy-supplying parts of the diets to keep the NE value constant among the dietary treatments at the same time [46, 51]. Therefore, the gain to feed ratio was equal with the caloric efficiency in these experiments. In many other studies, the NE to SID Lys ratio was kept constant among the dietary treatments when formulating treatment diets [12, 28, 47]. There is still no evidence showing which experimental design is more accurate in validation. The caloric efficiency can combine with other techniques, e.g. meta-analysis, to validate the NE content of ingredients as well as the prediction equations for growth performance established based on meta-analysis [49].

However, the actual NE value of the ingredients, especially the high-fiber ingredients, rely on the inclusion level of the ingredients in diets [2]. It was emphasized that the NE value of ingredients can be greatly affected at a high inclusion level in diets [48]. Furthermore, it is more common that no significant difference were observed among dietary treatments using the caloric efficiency approach, especially when the number of replications is insufficient. Under such conditions, it is difficult to conclude that the assigned energy value is accurate, because we can only infer that the value is not wrong from statistical point of view. As a result, there are some drawbacks to use the caloric efficiency method to validate the energy content of ingredients. In a more recent study, the target NE values of corn DDGS were compared with the estimated NE contents of DDGS based on the NRC growth model. Prediction error and bias between these two datasets were calculated and used to validate the target NE values [52]. This approach avoids to use caloric efficiency in energy content validation, but the choice of an appropriate model is vital to get convincing results.

## Conclusions and perspectives for future

The NE system provides a good foundation to increase the utilization of alternative feedstuffs in swine diets. However, research on NE is time-consuming, expensive, and complex and depends heavily on methodology. This review summarizes the NE research in swine conducted at MAFIC from methodological aspect. The net energy requirements for maintenance were estimated in MAFIC using both the fasting heat production method and the nonlinear regression method. The NE value of feedstuffs were determined using indirect calorimetry through standard experimental procedure in MAFIC, and the prediction equation from INRA (NE = (0.7 × DE) + [(1.61 × EE) + (0.48 × Starch) - (0.91 × CP) - (0.87 × ADF)]/1000 × 4.184) can be used to predict NE in situations where calorimeters are not available. Although with drawbacks, caloric efficiency is still the most popular parameter to validate the estimated NE content. In the future, new criteria established on biochemical mechanisms can be used to describe the maintenance condition for pigs. NE prediction equations based on individual ingredients should be established to improve the accuracy of prediction. More generally accepted approach is needed to further validate the estimated NE values of feedstuffs.

## Abbreviations

ADFI: Average daily feed intake
ADG: Average daily gain
BW: Body weight
DE: Digestible energy
DM: Dry matter
FHP: Fasting heat production
GE: Gross energy
HI: Heat increment
HP: Heat production
k_g_: Efficiencies of energy utilization for growth
k_m_: Efficiencies of energy utilization for maintenance
ME: Metabolizable energy
ME_m_: Metabolizable energy requirements for maintenance
N: Nitrogen
NE: Net energy
NEE: Net energy efficiency
NE_m_: Net energy requirements for maintenance
RE: Retention energy
RE_L_: Retention energy as lipid
RE_P_: Retention energy as protein
THP: Total heat production
